# Defining health and lifestyle characteristics of the age 50+ population: cluster analysis of data from the PROTECT study

**DOI:** 10.3389/fpubh.2025.1528952

**Published:** 2025-07-18

**Authors:** Emily Kontaris, Ian Wakeling, Helen Brooker, Anne Corbett, Clive Ballard, Dag Aarsland, Anne Churchill

**Affiliations:** ^1^Health and Well-being Centre of Excellence, Givaudan UK Limited, Ashford, United Kingdom; ^2^Qi Statistics Limited, West Malling, United Kingdom; ^3^Ecog Pro Limited, Bristol, United Kingdom; ^4^Department of Health & Community Sciences, University of Exeter, Exeter, United Kingdom; ^5^Institute of Psychiatry, Psychology and Neuroscience, King's College London, London, United Kingdom

**Keywords:** successful aging, life events and contexts, life course and developmental change, multidimensional approach, healthy aging

## Abstract

**Introduction:**

The proportion of older people in the world is increasing and evidence suggests that older adults interact differently with products. Understanding this change is necessary to develop products that satisfy this cohort’s needs. Chronological age is typically used to segment older consumers however, given the diversity of ageing, a multi-dimensional approach considering other factors contributing to this behavior change is important. Using data from the PROTECT study in the UK, this research aimed to identify clusters of older people with distinct characteristics and investigate whether chronological age was fundamental in defining these groups.

**Methods:**

Twelve variables, covering measures related to physical capabilities, mental health and lifestyle choices, were derived from the baseline questionnaire data from the PROTECT study and subjected to a k-means cluster analysis. Subsequent analyses investigated the association between participants’ cluster membership and other key variables.

**Results:**

Cluster analysis identified 8 unique clusters of older adults differentiated on factors such as physical health (physical activity, pain, BMI and sleep quality), mental health (cognitive decline, depression and anxiety) and lifestyle (social events, puzzle and technology use and vitamin intake). Age was considered to be an important contributory factor to some clusters however did not explain all differences observed between the groups.

**Discussion:**

Our findings indicate that in addition to chronological age, health and lifestyle variables are important in defining the unique characteristics of different clusters of those in the 50+ cohort. Future research should consider the multi-dimensional nature of ageing when conducting research with older consumers.

## 1 Introduction

Development of successful consumer products relies on understanding the potential market and in particular the characteristics of the consumer population that define their purchasing decisions. Companies will use a raft of sophisticated market research techniques to help them understand the values and priorities, preferences and obstacles, habits and experiences of the consumer cohort they are interested in. Demographic and social measures used to classify individuals within a population often include, age, household income, marital status, employment, education, number of dependent children, technology or social media use, shopping venue of choice and specific product usage which have been shown to relate to product choice (1).

The number of older individuals worldwide is increasing. According to data from the United Nations (2022) World Population Prospects, by 2050, one in four persons living in Europe and North America could be aged 65 or older (2). On average older consumers have relatively large disposable incomes and many of them are active consumers with plenty of free time (3). Consequently, there is increased interest in developing products that will be attractive to this cohort and a resulting demand to better understand the characteristics of the members of this population. Consumer products companies have shifted their focus from virtually exclusive consideration of the population under the age of 45 to also include those over 55 (4). Defining the key characteristics of this cohort of consumers and how these influence their shopping habits has become a research necessity.

When examining the behavior of the older consumer and defining segments with differing needs within the group the most easily accessible classification is by chronological age. The threshold age for older consumers is still debated but commonly 65 years old is used as the age when consumers enter this group (5). This is the age at which individuals typically retire which could explain the choice of this threshold (6). A review by Tongren (7) also confirmed that most published articles used this threshold although several studies do use lower ages such as 50 or 55 years old (8, 9).

An alternative approach is to classify consumers by generation or cohort (10). These cohorts span seven different generational groups from The Greatest Generation (born 1901–1927) to Generation Alpha (born 2013–2025) and remain consistent throughout the individual’s lifespan, with members of the same group often displaying similar consumer behaviors (11).

When considering how people interact with products, ageing factors such as sensory acuity and cognitive and physical capabilities have been shown to be important in determining their satisfaction and pleasure in using the products that they buy. However, the rates at which these factors decline for different individuals as they age vary enormously (12). There is still an on-going debate about how to best define ageing in the context of older consumers (13) and the high levels of diversity in rates of decline has created concern over the suitability of chronological age or age cohort for defining older consumer groups (10).

Other approaches have been suggested by researchers such as Barak and Schiffman (14) who proposed cognitive age as an alternative, which is defined as the self-perceived (or subjective) age of a person (15). Catterall and Maclaran (6) suggested that attitudes towards ageing are also an important consideration, while Kuppelweiser and Klaus (13) argued that the Future Time Perspective, determined by the time that people believe they have left to live, was more appropriate.

The Lancet Commission’s reports of 2020 and 2024 (16, 17) highlighted the importance of an individual’s engagement with controllable risk factors that can modify susceptibility to the on-set of dementia. Fourteen modifiable risk factors were listed in the 2024 report including level of education, hypertension, hearing impairment, smoking, obesity, depression, physical inactivity, diabetes, low social contact, excessive alcohol consumption, traumatic brain injury, air pollution, untreated vision loss and high LDL cholesterol. Although many of these factors cannot be influenced by the individual the authors concluded that there is a huge potential for people to reduce their dementia risk and, the evidence suggests, by doing so they will increase the number of healthy years of life and compress the duration of ill health for those with dementia, if over the course of their life they change their behavior relevant to the risk areas.

More recently the multidimensional nature of subjective ageing and specifically the concept of subjective awareness of age-related change (AARC) has been introduced (18). Questionnaires have been developed (AARC-50 and AARC-10) that measure the positive and negative ageing experiences across five behavioral domains (health and physical functioning, cognitive functioning, interpersonal relations, social-cognitive and social-economic functioning, lifestyle and engagement). Use of these multidimensional domains extends the ideas previously proposed that cover self-perception of cognitive age and attitudes towards ageing and claim to include the areas of an adult’s life that give rise to subjective ageing experiences. Measurement across these domains has been shown to be a better predictor of health and well-being over and above unidimensional measures of subjective ageing (19, 20). The innovative strength of these questionnaires is the measurement of both positive and negative perceptions of ageing, described as gains or losses, which describe different aspects of self-perceived ageing. Research has shown that positive perceptions of ageing appear to serve as a protective factor in ageing, for instance in the face of a serious health condition (21), whereas negative perceptions are predictive of negative outcomes (22).

Taking into account the diverse perspectives of ageing, in addition to the consideration of modifiable risk factors, makes identifying and targeting the older consumer group for product testing a complex task. It is therefore important to consider how different measures of the ageing experience together with more traditional demographic measures could be relevant in describing this older consumer group and identifying which factors are important to understand their different needs and interests.

Using data from the PROTECT study in the UK, the primary aim of this research was to identify and cluster different groups of people from the cohort based on their similar characteristics and define unique features of the groups. A secondary aim was to explore whether chronological age was an important factor in classifying the groups or whether other variables were considered to be equally or more important in defining the different group characteristics.

## 2 Methods

### 2.1 Participants and procedure

This study used baseline data from the PROTECT study,^1^ collected between November 2014 and April 2021. The PROTECT study aims to explore how factors such as genes, lifestyle and health change with age, particularly how these factors can influence cognition. Participants from the PROTECT study were UK residents, English speakers and aged 50 years and over, with access to the internet and no clinical diagnosis of dementia. The study was advertised through national publicity and existing cohorts of older adults and approved by London Bridge NHS Research ethics Committee and Health Research Authority (reference: 13/LO/1578).

The baseline data used in this study included a total of 3,740 participants (Females = 2,746, Males = 994; age range: 50–94 years). A full breakdown of the demographics of the sample can be found in Table 1.

**Table 1:** Summary of demographic characteristics of study sample.

Demographic variable	Sample (%)
Age (*n* = 3,737)	
50–54 years	14.2
55–59 years	19.8
60–64 years	22.3
65–69 years	20.7
70 + years	23.0
Sex (*n* = 3,740)	
Male	26.6
Female	73.4
Ethnicity (*n* = 3,144)	
White	98.1
Other ethnic background	1.9
Marital status (*n* = 3,140)	
Single	6.0
Married	68.6
Civil Partners/Co-habiting	7.1
Separated/Divorced	12.4
Widowed	5.9
Education (*n* = 3,144)	
Secondary	10.3
Post-Secondary	10.3
Undergraduate	32.8
Postgraduate	25.3
Vocational	21.4
Employment status (*n* = 3,135)	
Employed full-time	18.3
Employed part-time	16.2
Self-employed	9.4
Retired	53.2
Unemployed	2.9

### 2.2 Measures

#### 2.2.1 Demographics

Measures comprised age, sex, ethnicity, marital status and employment status.

#### 2.2.2 Daily living difficulties

Measured by the Instrumental Activity for Daily Living (IADL) (23), a 14-item questionnaire where participants are asked to rate 7 activities on the level of help needed to perform the task and how difficult they find these to complete.

#### 2.2.3 Mild behavior impairment (MBI)

The Mild Behavioral Impairment Checklist (MBI Self) (24), a 34-item questionnaire, which measured whether there have been any changes in participants’ behavior. Participants rated items on presence and severity of change in behavior.

#### 2.2.4 Cognitive decline

Cognitive decline was assessed using the Informant Questionnaire on Cognitive Decline in the Elderly (IQCODE) (25), a 16-item questionnaire, where participants rated how they are now compared to 10 years ago.

#### 2.2.5 Lifestyle

Comprised various dimensions relating to participants’ lifestyle habits. For *Physical Activity* participants indicated how many times they had taken part in physical activity (in the past month) that lasted 20 min and left them out of breath. For *Puzzles,* participants rated the frequency in which they completed puzzles, as well as the frequency of use of *Technology*. Participants also indicated their *Vitamin Intake* from a pre-defined list of 11 vitamins and supplements. Participation in *Social Events* was measured by the CHAMPS physical activity questionnaire for older adults (26) where participants specified the frequency in which they completed certain activities, specifically non-sporting events outside of the home.

#### 2.2.6 Sleep

Sleep Quality was assessed using the St Mary’s Hospital Sleep Questionnaire (27), specifically for the question “How would you rate how well you sleep?”.

#### 2.2.7 Mental health

Measures of mental health were based on participants’ responses to the Generalised Anxiety Disorder Assessment (GAD-7) (28) and the Patient Health Questionnaire (PHQ9) (29).

#### 2.2.8 Physical health

Measures of physical health were based on participants’ responses to several questions including their height and weight to calculate Body Mass Index (BMI), *Pain* where they were asked whether they experience pain in their daily lives and if so how much the pain interferes with specific scenarios, as well as an indication of their diagnosis of any medical conditions.

### 2.3 Cluster analysis variables

A total of 12 variables were derived from the questionnaire data to provide an overview of each subject’s general wellbeing and quality of life (Table 2). Most of the variables included are composite variables however a few variables thought to be important in their own right were used unmodified. It is important to note that over the course of the study questionnaires were added at various points in time, with participants also joining the study at different times and as a result not all participants completed all questionnaires. Therefore, the 12 variables were selected from the most frequently administered questionnaires.

**Table 2:** Overview of variables used in cluster analysis and details of how these were computed.

Variable name	Calculation
Daily living difficulties	A single score summarizing the 14 questions in the IADL questionnaire. For each of 7 common activity types (meals, housework, finances, medications, telephone, shopping and transport), the perceived level of difficulty and degree of help needed to perform the activity are scored on a 0–2 point scale. Difficulty and help scores are first multiplied together and then averaged giving a final score in the range zero to 4
Mild behavior impairment	A single average score per subject summarising the severity ratings on a 0–3 scale of 34 issues that cover behavioral and memory loss problems
Cognitive decline	A single average score per subject summarising the self-assessed severity of the 16 items from the IQC memory and reasoning decline questionnaire. High average scores on the 5-point scale, are associated with a condition that has worsened when compared to 10 years ago
Body mass index (BMI)	Body mass index derived from the subject’s weight in pounds and height inches calculated as 703*weight/height2
Pain	A pain score which is zero if the subject answered that they were free from pain that interferes with their day-to-day life, otherwise the score is the average of four pain ratings (0–4 point scale) that measure pain associated with common activities
Sleep quality	The response to the question “how would you rate how well you sleep?” measured on a six-point scale from very badly (1) to very well (6)
Puzzles and technology use	The average frequency of use score from 7 questions regarding subject engagement with mental games (word/number puzzles and computer training) and computer technologies
Physical activity	Frequency of periods of physical activity lasting at least 20 min within the last month, measured on a 5-point scale from None (0) to More than 20 Times (4)
Social events	The average of 6 variables which measure the frequency with which subjects attend different types of non-sporting events outside of the house. Each variable is measured on a 3-point scale from never (0) to twice or more per week (2)
Vitamin intake	The total number of dietary supplements taken by the subject from a list of 11
Anxiety	Anxiety as measure by the GAD7 questionnaire
Depression	Depression as measured by the PHQ9 questionnaire

### 2.4 Data analysis

For the 12 variables selected a total of 3,740 participants with complete data were used for cluster analysis (30). To prevent the different measurement scales influencing the analysis, each variable was standardized to zero mean and unit variance prior to analysis. A k-means cluster analysis was then performed on the data using XLSTAT (2022) software (31). The Euclidean metric was used with the determinant of the pooled within group covariance matrix as the clustering criterion. K-means solutions with between 5 and 10 clusters were examined, each one being the best solution found from 100 runs with different random starting seeds. An 8 cluster solution was selected as the optimal solution as this resulted in the most evenly distributed cluster sizes and enabled a clearer interpretation of the clusters.

Following cluster analysis, cross-tabulations were performed to assess the degree of association between participants’ cluster membership and various other variables. The XLSTAT module for testing contingency tables was used to compute standard chi-squared tests, and, where significant, post-hoc tests of cell significance using Fisher’s exact test (32).

## 3 Results

### 3.1 Cluster summary

**Figure 1:**
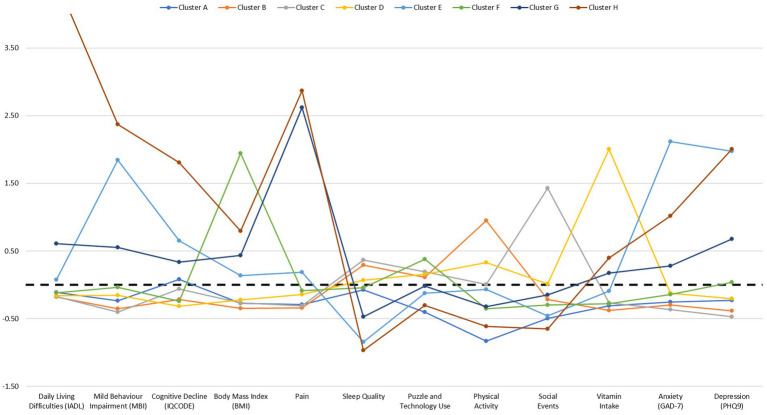
Graphical summary of 8 cluster solution and associated cluster centroids. Dotted line at zero indicates average scores. Score for Cluster H and Daily Living Difficulties (IADL) variable is large and therefore not shown on the graph to improve visibility of other data points.

A summary of the 8 cluster solution and the cluster centroids associated with each of the variables included in the analysis is shown in Table 3. Cluster centroids are highlighted for variables considered to be most important to the cluster. Figure 1 presents a graphical overview of the clusters.

**Table 3:** Summary of 8 cluster solution and associated cluster centroids.

Cluster	A	B	C	D	E	F	G	H
*N*	872	862	646	415	333	320	255	37
%	23.3	23.0	17.3	11.1	8.9	8.6	6.8	1.0
Daily Living Difficulties (IADL)	−0.11	−0.18	−0.18	−0.16	0.07	−0.11	0.61	**7.87**
Mild Behavior Impairment (MBI)	−0.24	−0.35	−0.40	−0.15	**1.84**	−0.03	0.55	**2.37**
Cognitive Decline (IQCODE)	0.08	−0.22	−0.06	−0.32	0.65	−0.24	0.33	**1.81**
Body Mass Index (BMI)	−0.27	−0.35	−0.27	−0.22	0.13	**1.94**	0.43	0.80
Pain	−0.29	−0.34	−0.31	−0.14	0.19	−0.09	**2.62**	**2.87**
Sleep Quality	−0.08	0.29	0.37	0.07	**−0.84**	−0.04	−0.47	**−0.97**
Puzzle and Technology Use	−0.40	0.11	0.19	0.16	−0.12	0.38	−0.02	−0.30
Physical Activity	**−0.83**	**0.95**	0.01	0.33	−0.07	−0.35	−0.32	−0.61
Social Events	−0.50	−0.22	**1.43**	0.01	−0.46	−0.29	−0.15	−0.65
Vitamin Intake	−0.31	−0.38	−0.27	**2.01**	−0.10	−0.28	0.17	0.40
Anxiety (GAD-7)	−0.26	−0.30	−0.36	−0.13	**2.12**	−0.15	0.28	**1.02**
Depression (PHQ9)	−0.23	−0.38	−0.47	−0.20	**1.98**	0.04	0.68	**2.00**

Cluster centroids that are +/− 0.8 are indicated in bold.

The majority of participants (63.6%) were grouped into the first three clusters, Cluster A: Low physical activity (*n* = *872*; 23.3%), Cluster B: High physical activity (*n* = 862; 23.0%) and Cluster C: High participation in social events (*n* = 646; 17.3%). Nearly all of the other participants were categorised into one of the subsequent four clusters; Cluster D: High vitamin intake (*n* = 415; 11.1%), Cluster E: High anxiety and depression, low sleep quality and mild behavior impairment (MBI) (*n* = 333; 8.9%), Cluster F: High BMI (*n* = 320; 8.6%) and Cluster G: High pain (*n* = 255; 6.8%). The final cluster was the smallest, yet significant, cluster identified, Cluster H: High daily living difficulties, high MBI, pain, anxiety, depression, low sleep quality and cognitive decline (*n* = 37; 1.0%).

### 3.2 Cluster profiles

Wherever there was a significant chi-squared test (*p <* 0.05) on the contingency table, formed by cross-tabulating cluster membership with a variable of interest, associations within each cluster are reported using Fisher’s exact test. Percentages reported are a comparison to the percentage expected from the population sampled. Key findings of associations between variables of interest and cluster membership are reported in the cluster profile summaries below. Full details of all significant associations can be found in Supplementary Tables S1–S3.

#### 3.2.1 Cluster A

This group was characterised by low physical activity and comprised 6.3% more Males (*p* < 0.001) and 2.0% fewer individuals who were single (*p* = 0.009). For participation in hobbies, this cluster included 6.2% more of those who never took part in arts and craft, such as woodworking, needlework, drawing or other arts and crafts activities (*p* < 0.001).

#### 3.2.2 Cluster B

This cluster was characterised by high physical activity, comprising more of the younger age groups, specifically 4.5% more 50–54 year olds (*p* < 0.001) and 3.9% more 60–64 year olds (*p =* 0.002). Additionally, this cluster comprised 6.9% more of those who were married (*p* < 0.001) and 5.0% more of those employed full-time (*p =* 0.000).

Cluster B also consisted of 11.0% more individuals who drank alcohol at least weekly (*p* < 0.001). In addition, this cluster comprised 2.9% more of those who read twice or more a week (*p =* 0.001) and 3.7% more of those who carried out heavy gardening activities (digging or raking) twice or more per week (*p =* 0.001).

#### 3.2.3 Cluster C

This cluster was characterised by high participation in social events outside of the house and comprised more of the older age groups, specifically 5.8% more 65–69 year olds (*p =* 0.000) and 11.1% more of those 70 years and older (*p* < 0.001). This cluster also consisted of 3.9% more of those who were widowed (*p* < 0.001), as well as 21.7% more retired individuals (*p* < 0.001). Additionally, this group included 5.5% more individuals educated to undergraduate degree level (*p =* 0.002).

Furthermore, this group comprised 3.6% more individuals who drank alcohol at least weekly (*p =* 0.04) and consisted of more of those who participated in various hobbies at least once or twice or more a week. This included 4.0% more of those who took part in arts and crafts once a week (*p =* 0.004), 4.8% more individuals who played a musical instrument twice or more a week (*p <* 0.001), 5.3% more individuals who read twice or more a week (*p <* 0.001) and 2.6% more individuals who participated in heavy gardening twice or more a week (*p =* 0.046).

#### 3.2.4 Cluster D

This cluster was primarily characterised by high vitamin intake, comprising 7.1% more females (*p =* 0.000) as well as 5.5% more of those educated to post-graduate level (*p =* 0.017). Cluster D also consisted of 2.4% more of those diagnosed with osteoporosis (*p =* 0.028) and 3.3% more of those diagnosed with asthma (*p =* 0.041). This group also included 5.0% more individuals who participated in light gardening activities, such as watering plants (*p =* 0.033) twice or more per week.

#### 3.2.5 Cluster E

This cluster was characterised by low sleep quality, high anxiety and depression and mild behavior impairment (MBI). This cluster included more individuals in the younger age groups, specifically 9.3% more 50–54 year olds (*p <* 0.001) and 4.6% more 55–59 year olds (*p =* 0.031). Additionally, this group comprised 6.7% more of those who were single (*p <* 0.001) as well as 4.6% more of those educated to secondary level (*p =* 0.012) and 3.9% more of those educated to post-secondary level (*p =* 0.035).

Furthermore, this cluster included 9.0% more individuals employed full-time (*p =* 0.000), 7.0% more individuals employed part-time (*p =* 0.002) as well as 5.3% more unemployed (*p <* 0.001). This group comprised 4.1% more individuals who had previously experienced a head injury where they lost consciousness (*p =* 0.045).

#### 3.2.6 Cluster F

This cluster was identified as the High BMI group, comprising more of the youngest age group, specifically 4.6% more 50–54 year olds (*p =* 0.019). This group also included 5.8% more of those who were single (*p =* 0.000), 3.8% more individuals educated to post-secondary level (*p =* 0.043) and 9.9% more individuals employed full time (*p <* 0.001).

For physical health variables, this cluster comprised 14.0% more individuals with a diagnosis of high blood pressure (*p <* 0.001), 3.9% more of those diagnosed with diabetes (*p =* 0.001) and 5.5% more of those with a diagnosis of an arthritic condition (*p =* 0.014). Additionally, this cluster included 5.2% more individuals who took part in arts and crafts twice or more a week (*p =* 0.024), 15.6% more of those who never conducted heavy gardening (*p <* 0.001) and 5.0% more who never conducted light gardening activities (*p =* 0.017).

#### 3.2.7 Cluster G

This cluster was mainly characterised as the high pain group, comprising 4.9% more individuals educated to secondary level (*p =* 0.020), 5.8% more of those who stated vocational as their highest educational level (*p =* 0.039) and 10.0% more individuals who were retired (*p =* 0.002).

For physical health, this cluster consisted of 12.5% more individuals diagnosed with high blood pressure (*p <* 0.001), 3.9% more individuals with a diagnosis of heart disease, heart attack or angina (*p =* 0.008), 3.1% more individuals diagnosed with diabetes (*p =* 0.018), 31.7% more of those diagnosed with an arthritic condition (*p <* 0.001), 4.8% more individuals with a diagnosis of osteoporosis (*p =* 0.001) and 5.0% more individuals diagnosed with asthma (*p =* 0.019). Additionally, the group comprised 6.0% more individuals who had hearing problems (*p =* 0.032) and 5.3% more of those who have previously experienced a head injury where they lost consciousness (*p =* 0.023).

This group also included 4.2% more of those who stated they drank alcohol less than once per month (*p =* 0.045) and 6.8% more of those who stated they never drank (*p =* 0.000). For participation in various hobbies, this cluster comprised 5.4% more of those who took part in arts and crafts at twice or more per week (*p =* 0.034), 2.9% more of those who played a musical instrument once per week (*p =* 0.049) as well as 10.1% more of those who never conducted heavy gardening (*p =* 0.001).

#### 3.2.8 Cluster H

This cluster was characterised by very high daily living difficulties, high MBI, BMI and anxiety and depression as well as a decline in cognition and poor sleep quality. This cluster comprised 16.6% more individuals educated to post-secondary level (*p =* 0.013) and 20.2% more individuals who were unemployed (*p <* 0.001).

This group consisted of 15.0% more of those diagnosed with high blood pressure (*p =* 0.046), 11.1% more individuals with a diagnosis of heart disease, heart attack or angina (*p =* 0.011), 23.1% more individuals diagnosed with diabetes (*p <* 0.001), 39.0% more individuals diagnosed with an arthritic condition (*p <* 0.001) and 25.2% more of those diagnosed with asthma (*p <* 0.001). Additionally, this group included 33.2% more of those who have problems with their hearing (*p <* 0.001) and 23.9% more individuals who have previously experienced a head injury where they lost consciousness (*p =* 0.000).

This cluster also consisted of 21.7% more individuals who drank alcohol less than once a month (*p =* 0.001) and 28.9% more of those who never drank (*p <* 0.001). For participation in hobbies, this group included 15.8% more of those who took part in arts and crafts twice or more per week (*p =* 0.027), 15.5% more of those who never read (*p =* 0.000) and 35.0% more of those who stated they never conducted heavy gardening (*p <* 0.001).

## 4 Discussion

This research identified 8 distinct clusters of older people using baseline data from the PROTECT study and classified these individuals based on their similar characteristics. Clusters were differentiated on factors such as physical health, mental health and lifestyle.

The two largest clusters differed according to physical activity levels, with Cluster A characterised by low physical activity and Cluster B characterised by high physical activity. Notably, Cluster A comprised significantly more males than expected, although caution should be taken with this finding as in general the proportion of male participants in this study was much lower than female participants. Cluster B comprised a majority of younger age groups and those who were employed full time. The relationship between age and physical activity has been observed in previous studies suggesting that in general physical activity declines as individuals age (33). Additionally, the association of this cluster with age and employment status is consistent with the average age of retirement in the UK (34). Interestingly, this cluster consisted of more individuals who reported to drink alcohol at least weekly. Alcohol consumption could be driven in part by the age distribution of this cluster as studies have suggested that alcohol consumption may decrease with age, although factors such as country, gender, socio-economic status and health status also play a significant role with regards to the amount of alcohol consumed (35, 36). Alternatively, there is some evidence for a link between increased physical activity and increased or moderate alcohol consumption although many of these studies tend to focus on younger populations (37). Indeed, some studies have found that when age, alongside gender, is taken into account this association is no longer significant (38).

The third largest cluster, Cluster C, was characterised by a high participation in social events and comprised more of those from the older age groups, as well as more individuals who were retired and widowed. Additionally, this cluster was characterised by individuals who participated in various hobbies several times a week, such as arts and crafts, playing a musical instrument and reading. The positive association with retirement and participation in leisure activities has been found in previous research with the suggestion that this could be due to individuals substituting their previous job role with other activities to supplement their time (39). Although this study also found an inverse relationship between leisure activity participation and non-married status which is inconsistent with our findings as this cluster comprised more individuals who were widowed.

The subsequent clusters consisted of fewer of the total sample however were clearly differentiated across various domains. Cluster D was defined as the high vitamin intake group and comprised of more of those educated to post-graduate level as well as more females. This finding is corroborated by previous research with several studies finding a consistent link between the use of vitamins or dietary supplements, having a higher education level and being female (40). Cluster E was characterised by poor sleep quality, higher scores on anxiety and depression questionnaires as well as higher mild behavioral impairment (MBI). This cluster also contained more of those from the younger age groups, those who were single and employed full-time. The association between poor sleep quality, anxiety and depression has been found in previous studies with the suggestion of a bi-directional relationship between these variables. For example, a study by Jansson-Fröjmark and Lindblom (41) found that anxiety and depression at baseline predicted subsequent reports of insomnia and insomnia at baseline predicted subsequent reports of anxiety and depression.

Cluster F was characterised as the high BMI group which was made up of more of those in the youngest age group. Additionally, this cluster contained more individuals with diagnoses of physical health conditions such as high blood pressure, diabetes or an arthritic condition as well as more of those who took part in less active hobbies such as arts and crafts and fewer of those who took part in more active hobbies such as gardening. This echoes public health information highlighting the link between being overweight or obese and various physical health conditions such as cardiovascular disease, diabetes and musculoskeletal disorders (42), as well as the association between sedentary behavior and higher BMI (43). Cluster G was classified as the high pain group with this cluster consisting of more individuals with diagnoses of various physical health conditions such as high blood pressure, heart disease, heart attack or angina, diabetes, an arthritic condition, osteoporosis and asthma. Indeed, studies have shown an association between reported pain severity and several health conditions such as cardiovascular disease (44) and osteoarthritis (45). Additionally, this cluster contained more individuals who either abstained from alcohol or consumed alcohol infrequently. This result is in line with findings from Moos et al. (46), who found that the occurrence of medical conditions and physical symptoms predicted an overall reduction in the frequency of alcohol consumption. This was likely due to an increase in awareness from participants of their poorer physical health and potential adverse effects of alcohol, rather than a negative interaction between medication use and alcohol consumption. However, this study also found that those who experienced greater health burden were more likely to have problems with alcohol consumption later on. Indeed, other studies have suggested that reports of recent pain were linked to increased frequency in alcohol intake (47) therefore the relationship between alcohol consumption and health status is multifaceted.

Lastly, the smallest cluster found in the analysis was Cluster H, which showed clear differentiation from the other clusters based on several variables. Specifically, this cluster was characterised by very high daily living difficulties, high MBI, BMI, anxiety, depression as well as a decline in cognitive facets (such as memory and reasoning) and poor sleep quality. Additionally, this cluster contained more of those who had been diagnosed with various physical health conditions, as well as more individuals who had reported that they had previously had a head injury where they lost consciousness. Research has suggested a possible link between mild behavioral impairment symptoms and prior head injury, although this association was specific to MBI domains of affective dysregulation and impulse dyscontrol (48).

With regards to the secondary aim of the study, our findings indicate that chronological age is an important characteristic for some clusters, specifically clusters B, C, E and F, however this variable did not explain all differences observed between the different clusters and ultimately the groups were characterised by other contributory factors, such as physical health, mental health and lifestyle. This finding suggests that age alone is insufficient to characterise the older population and supports previous research highlighting the multi-dimensional nature of ageing (10). Importantly, this emphasises the need for market research to recognise the diversity of the older consumer group and implement strategies for segmenting older consumers using a gerontological approach, considering biological, social, cognitive and cultural factors associated with ageing (49). Indeed, research has started to take this into account with some studies presenting a multivariate approach to older consumer segmentation with recommendations on how to specifically market to these distinct groups (3) or specifically highlighting key changes in older consumers’ lifestyles and behaviors which could have significant implications for business marketing strategies (49), although these approaches should become more commonplace to enable companies to effectively target this consumer group.

It is important to note some limitations of this study. Participants were passively recruited for the PROTECT study by advertisement and no attempt was made to balance the demographic structure. As a result, the sample population is biased towards white, highly educated, married females under the age of 70. Additionally, as the study is carried out online, and some tests are quite complex and time-consuming older participants could be discouraged from completing all sections of the study. The effect of this sample bias on the data structure is unknown and future attempts will be made to rectify this, however this study provides valuable insight into the characteristics important in classifying this age group. Future research could explore whether the cluster structure and defining characteristics found in this study still holds when including responses from a more diverse sample population. Additionally, the data used for this study was gathered from questionnaires from which the most complete dataset could be obtained and it is possible that other factors important for ageing measured by questionnaires not included in this analysis could provide additional insights into the characterisation of this population. Future studies could investigate whether the inclusion of other questionnaire measures, such as the AARC, would extend these results further.

Overall, this study has identified distinct groups of people aged 50 years and over, defined by different characteristics which cannot be solely attributed to chronological age. Previous research has highlighted that age-related factors can influence older consumers’ responses to products and services (11, 50). Specifically, differences in cognitive age can influence responses to products and retail offerings (51), older people assume their new roles as grandparents or retirees and develop new needs for products and services (11) and life events can affect attitudes of people experiencing them and may influence consumption patterns (52). Inclusion of these considerations in future research is important in providing a fuller understanding of potential consumer market choices particularly in the context of new product development.

## Data Availability

The data analyzed in this study is subject to the following licenses/restrictions: the analytic methods, data and results from this specific research activity will not be made publicly available due to ongoing research activities, however access to the UK PROTECT data could be made available upon request and following approval from the UK PROTECT steering committee. Requests to access these datasets should be directed to protect.data@exeter.ac.uk.

## Footnotes

^1^ https://www.protectstudy.org.uk
