# Supplementary material for: Defining health and lifestyle characteristics of the age 50+ population: cluster analysis of data from the PROTECT study
Source: Front Public Health. 2025 Jul 18;13:1528952. doi: 10.3389/fpubh.2025.1528952 (PMC12313484; doi:10.3389/fpubh.2025.1528952)
Supplement: Supplementary file 1 [file Data_Sheet_1.pdf]

Supplementary Table 1

Summary of percentages for each of the significant chi-square cross tabs for demographic variables

		Cluster (%)								Overall Sample (%)	χ^2	*p*
		**A**	**B**	**C**	**D**	**E**	**F**	**G**	**H**			
Sex											35.67	<0.0001
	Male	32.9 (>)	27.8	24.8	19.5 (<)	24.0	24.4	22.0%	32.4%	26.6		
	Female	67.1 (<)	72.2	75.2	80.5 (>)	76.0	75.6	78.0%	67.6%	73.4		
Age											197.77	<0.0001
	50-54 years	12.4	18.7 (>)	4.6 (<)	13.3	23.5 (>)	18.8 (>)	11.4%	21.6%	14.2		
	55-59 years	19.4	21.7	15.3	21.0	24.4 (>)	21.3	18.0%	10.8%	19.8		
	60-64 years	22.3	26.2 (>)	19.5	19.5	22.9	20.9	22.0%	21.6%	22.3		
	65-69 years	21.4	16.7 (<)	26.5 (>)	22.4	13.6 (<)	20.9	23.1%	24.3%	20.7		
	70+ years	24.6	16.6 (<)	34.1 (>)	23.9	15.7 (<)	18.1 (<)	25.5%	21.6%	23.0		
Marital Status											104.07	<0.0001
	Single	4.0 (<)	3.4 (<)	6.0	5.1	12.7% (>)	11.8 (>)	8.3	0.0	6.0		
	Married	70.6	75.5 (>)	65.6	69.6	58.2% (<)	62.4 (<)	66.8	57.7	68.6		
	Civil Partners/Co-habiting	8.4	6.5	5.1 (<)	5.7	9.7%	8.7	5.5	15.4	7.1		
	Separated/Divorced	12.0	9.9 (<)	13.5	14.0	14.6%	12.5	13.4	19.2	12.4		
	Widowed	5.0	4.7	9.8 (>)	5.7	4.9%	4.6	6.0	7.7	5.9		
Education											91.75	<0.0001
	Secondary	11.3	9.3	5.1 (<)	10.1	14.9 (>)	12.9	15.2 (>)	11.5	10.3		
	Post-Secondary	10.2	10.9	6.9 (<)	6.3 (<)	14.2 (>)	14.1 (>)	12.0	26.9 (>)	10.3		
	Undergraduate	33.0	33.7	38.3 (>)	33.1	27.6	31.9	22.1 (<)	26.9	32.8		
	Postgraduate	23.2	26.5	28.5	30.7 (>)	22.4	19.4 (<)	23.5	11.5	25.3		
	Vocational	22.3	19.6	21.3	19.7	20.9	21.7	27.2 (>)	23.1	21.4		
Employment Status											313.45	<0.0001
	Employed full-time	20.6	23.3 (>)	5.1 (<)	15.9	27.3 (>)	28.2 (>)	10.6 (<)	3.8	18.3		
	Employed part-time	14.1	18.5	11.4 (<)	18.3	23.2 (>)	19.8	12.4	7.7	16.2		
	Self-employed	9.1	10.4	8.1	10.5	8.6	10.3	8.8	7.7	9.4		
	Retired	53.5	46.3 (<)	74.8 (>)	53.3	32.6 (<)	37.0 (<)	63.1 (>)	57.7	53.2		
	Unemployed	2.6	1.5 (<)	0.5 (<)	2.1	8.2 (>)	4.6	5.1	23.1 (>)	2.9		

Note: For clusters A to H significance ($p<0.05$) is reported from Fisher's exact test and indicated by greater than (>) or less than (<) signs

Supplementary Table 2

Summary of percentages for each of the significant chi-square cross tabs for physical health variables.

		Cluster (%)								Overall Sample (%)	χ^2	***p***
		**A**	**B**	**C**	**D**	**E**	**F**	**G**	**H**			
Diagnosis of Medical Condition												
High Blood Pressure											86.37	<0.0001
	Yes	21.7%	17.2% (<)	22.0%	17.6% (<)	24.0%	36.9% (>)	35.3% (>)	37.8% (>)	22.8%		
	No	78.3%	82.8% (>)	78.0%	82.4% (>)	76.0%	63.1% (<)	64.7% (<)	62.2% (<)	77.2%		
Heart disease / Heart Attack / Angina											28.70	0.000
	Yes	5.7%	3.4% (<)	4.5%	4.1%	4.5%	7.5%	9.0% (>)	16.2% (>)	5.2%		
	No	94.3%	96.6% (>)	95.5%	95.9%	95.5%	92.5%	91.0% (<)	83.8% (<)	94.8%		
Diabetes											93.04	<0.0001
	Yes	3.7%	1.4% (<)	3.3%	2.4%	6.0%	7.8% (>)	7.1%	27.0% (>)	4.0%		
	No	96.3%	98.6% (>)	96.7%	97.6%	94.0%	92.2% (<)	92.9%	73.0% (<)	96.0%		
Arthritic condition												
	Yes	15.3% (<)	13.0% (<)	19.0%	23.4%	18.6%	25.9% (>)	52.2% (>)	59.5% (>)	20.5%	245.68	<0.0001
	No	84.7% (>)	87.0% (>)	81.0%	76.6%	81.4%	74.1% (<)	47.8% (<)	40.5% (<)	79.5%		
Osteoporosis											35.89	<0.0001
	Yes	4.6%	3.2% (<)	6.7%	7.7% (>)	6.0%	2.2% (<)	10.2% (>)	10.8%	5.3%		
	No	95.4%	96.8% (>)	93.3%	92.3% (<)	94.0%	97.8% (>)	89.8% (<)	89.2%	94.7%		
Asthma											43.33	<0.0001
	Yes	10.4% (<)	11.8%	9.4% (<)	15.9% (>)	15.0%	13.4%	17.6% (>)	37.8% (>)	12.6%		
	No	89.6% (>)	88.2%	90.6% (>)	84.1% (<)	85.0%	86.6%	82.4% (<)	62.2% (<)	87.4%		
Hearing problems											31.76	<0.0001
	Yes	29.6%	25.2% (<)	28.2%	27.7%	31.2%	29.4%	34.9% (>)	62.2% (>)	28.9%		
	No	70.4%	74.8% (>)	71.8%	72.3%	68.8%	70.6%	65.1% (<)	37.8% (<)	71.1%		
Head Injury											32.66	<0.0001
	Yes	16.1%	15.8%	12.8% (<)	17.8%	20.7% (>)	15.3%	22.0% (>)	40.5% (>)	16.6%		
	No	83.9%	84.2%	87.2% (>)	82.2%	79.3% (<)	84.7%	78.0% (<)	59.5% (<)	83.4%		

For clusters A to H significance ($p<0.05$) is reported from Fisher's exact test and indicated by greater than (>) or less than (<) signs

Supplementary Table 3

Summary of percentages for each of the significant chi-square cross tabs for lifestyle and hobbies variables

		Cluster (%)								Overall Sample (%)	χ²	*p*
		**A**	**B**	**C**	**D**	**E**	**F**	**G**	**H**			
Alcohol intake											170.00	<0.0001
	At least weekly	59.3 (<)	73.2 (>)	65.8 (>)	61.4	61.0	50.0 (<)	50.2 (<)	18.9 (<)	62.2		
	Less than once a week	15.7	12.3 (<)	16.6	16.6	15.9	19.4	16.5	8.1%	15.5		
	Less than once a month	14.8	8.8 (<)	10.2 (<)	12.8	15.3	21.6 (>)	17.6 (>)	35.1 (>)	13.4		
	Never	10.2	5.7 (<)	7.4	9.2	7.8	9.1	15.7 (>)	37.8 (>)	8.9		
Arts and crafts											45.94	<0.0001
	Twice or more per week	19.8	20.9	23.7	20.2	18.6	27.2 (>)	27.5 (>)	37.8 (>)	22.0		
	Once a week	14.1 (<)	17.9	22.1 (>)	20.2	18.6	17.5	19.2	16.2	18.1		
	Never	66.1 (>)	61.3	54.2 (<)	59.5	62.8	55.3	53.3 (<)	45.9	59.9		
Play musical instrument											46.15	<0.0001
	Twice or more per week	8.0	8.4	13.3 (>)	7.7	6.0	5.6	7.8	2.7	8.5		
	Once a week	5.2	5.2	7.3	7.2	4.2	3.4	8.6 (>)	0.0	5.7		
	Never	86.8	86.4	79.4 (<)	85.1	89.8 (>)	90.9 (>)	83.5	97.3	85.7		
Read											105.83	<0.0001
	Twice or more per week	89.2	93.4 (>)	95.8 (>)	91.3	82.0 (<)	88.1	87.1	70.3 (<)	90.5		
	Once a week	7.3	4.4 (<)	3.3 (<)	6.5	9.3 (>)	7.2	7.8	10.8	6.1		
	Never	3.4	2.2 (<)	0.9 (<)	2.2	8.7 (>)	4.7	5.1	18.9 (>)	3.4		
Heavy gardening											91.85	<0.0001
	Twice or more per week	13.4	18.9 (>)	17.8 (>)	17.3	11.7	10.3 (<)	10.6 (<)	2.7 (<)	15.2		
	Once a week	37.8	38.3	38.2	38.1	36.3	25.3 (<)	30.6	13.% (<)	36.1		
	Never	48.7	42.8 (<)	44.0 (<)	44.6	52.0	64.4 (>)	58.8 (>)	83.8 (>)	48.7		
Light gardening											31.38	0.005
	Twice or more per week	48.4 (<)	52.8	54.6	56.6 (>)	50.2	43.4 (<)	55.3	45.9	51.6		
	Once a week	32.2	30.7	31.7	28.4	30.3	34.1	25.1 (<)	32.4	30.9		
	Never	19.4	16.5	13.6 (<)	14.9	19.5	22.5 (>)	19.6	21.6	17.5		

Note: For clusters A to H significance ($p<0.05$) is reported from Fisher's exact test and indicated by greater than (>) or less than (<) signs
